# Secondary stroke prevention in atrial fibrillation: a challenge in the clinical practice

**DOI:** 10.1186/s12883-014-0195-y

**Published:** 2014-09-30

**Authors:** Christian Tanislav, Sonja Milde, Sabine Schwartzkopff, Nicole Sieweke, Heidrun Helga Krämer, Martin Juenemann, Björn Misselwitz, Manfred Kaps

**Affiliations:** Department of Neurology, Justus Liebig University, Klinikstrasse 33, 35392 Giessen, Germany; Federal Association of the AOK, Berlin Germany and Dresden International University, DIU (until March 2012), Dresden, Germany; AOK Division of the Federal State of Hesse, Eschborn Frankfurt, Germany; Geschäftsstelle Qualitätssicherung Hessen (GQH), Eschborn Frankfurt, Germany

**Keywords:** Stroke, Atrial fibrillation, New oral anticoagulants, Secondary prevention

## Abstract

**Background:**

Despite clear evidence for the effectiveness of oral anticoagulation (OA) in patients with atrial fibrillation (AF), there is evidence for the underutilisation of this therapy in the secondary stroke prevention. We therefore investigate the link between the use of OA in stroke patients with AF and favourable clinical outcome following the acute event.

**Methods:**

The study population was determined by identifying the overlap of two different databases: a stroke registry and claims data of a health insurance company. Baseline data originated from the registry; documented dementia and the prescriptions for OA were derived from the insurance database. Patients with AF, minor physical impairment, and evidence of more than 30 days without further hospitalisation within the subsequent 90 days after the acute event were selected for the analysis.

**Results:**

1828 patients were selected (mean age 77.6 years), 1064 patients (58.2%) were female. 827 patients (45%) received a prescription for OA. The following factors were independently associated with no prescription for oral anticoagulants: increased age (OR: 0.54, CI: 0.46-0.63; *P* < 0.0001), female sex (OR: 0.77, CI: 0.63-0.94; *P* < 0.011), worsening disability status at discharge (OR: 0.88, CI: 0.81-0.96; *P* < 0.006), and documented dementia (OR: 0.54, CI: 0.39-0.73; *P* < 0.001). Conversely, treatment in a neurological department was associated with prescription for OA (OR: 1.47, CI: 1.19-1.81; *P* < 0.003).

**Conclusions:**

In more than half of the patients with AF who suffered a stroke OA was not prescribed. The factors associated with reluctance in prescribing anticoagulants are increasing age, female sex, treatment at a non-neurological department, worsening disability, and dementia.

## Background

The high risk for recurrences in cardioembolic stroke due to atrial fibrillation (AF) renders secondary prevention mandatory [1]. Oral anticoagulation (OA) has been proven as an effective treatment for this condition [1-5]. In recent investigations new tools for risk stratification have been developed [6]. Accordingly, current guidelines for patients with cardioembolic stroke and AF recommend oral anticoagulant treatment [7]. A favourable risk/benefit ratio using this regimen was also proven in elderly patients [8]. However, registry data shows an underutilisation of this treatment option [9-11]. Lack of knowledge of current guidelines, concern for the risk of bleeding, socio-demographic factors (increased age, ability to cope with everyday tasks) and clinical factors (neurological deficits, dementia, and previous bleedings) are suspected to influence the low rates in the usage of OA in the clinical practice.

In this study, we investigated factors which might have impact on the decision to use OA in stroke patients with AF with a favourable clinical outcome after the acute event. For this reason, we analysed prescriptions documented nationwide in an insurance dataset in the Federal state of Hesse, Germany, together with baseline data, derived from a large comprehensive stroke registry.

## Methods

The presented study is based on data generated by the study of two different databases and where patient information records overlapped:

1. Registry data (2004–2010) of the Institute of Quality Assurance Hesse (Geschäftsstelle für Qualitätssicherung, GQH) [12]. The GQH database is an obligatory nationwide hospital-based registry that covers more than 95% of all ischemic strokes, TIA and intracerebral haemorrhages in a community of more than 6 million inhabitants of Hesse, Germany [13]. The GQH-data include details of acute inpatient treatment, as well as factors proved to be relevant for the course and the prognosis of stroke [12,13].
2. Claims data from a nationwide statutory health insurance company in Germany (AOK Hessen). The insurance database includes date of death and all billed services, as well as services provided by nursing care insurance. Insurance data between 2005 and 2007 were available for the analysis.

### Ethical issues

In Germany the acquisition of data for quality assurance reasons is regulated by law and implemented as a guideline, which is elaborated by the Federal Joint Committee for hospital quality assurance in accordance with Volume V of the Social Insurance Code (§137 SGB V and §135a SGB V). Based on this regulation the Hesse State Hospital Law contains a provision that allows the GQH to record such data legally. The publication of aggregate quality assurance data has also been cleared with the Hesse Data Protection Commissioner, so no data protection problem arises here either [13].

The protocol of the present study was reviewed and approved by the ethical committee of the medical faculty of the Justus Liebig University Giessen.

### Patient selection

Patients ≥ 18 years of age with an index event consistent of stroke or transient ischemic attack, diagnosed atrial fibrillation, a minimal physical impairment (modified Rankin scale (mRS) ≤3 on discharge) and direct discharge after acute treatment or referral subsequently to a rehabilitation facility were identified from the stroke registry. Among these patients, individuals affiliated to the insurance company were identified within the dataset in a pseudonymous manner according to the following matching criteria: sex, date of birth, date of admission and admitting hospital. Patients with evidence of at least a 30-day period free of additional hospitalisation within the 90 days following the initial acute treatment were considered for the analysis.

### Parameters and outcome measurements

For comparing baseline characteristics the following parameters were considered: age, sex, ischaemic stroke versus transient ischemic attack (TIA), previous stroke, vascular risk profile (hypertension and diabetes), disability status on discharge as assessed by mRS, treatment in a neurological department and comorbidities occurring during hospitalisation.

To determine the therapeutic management for secondary stroke prevention we analysed prescriptions for oral anticoagulants (including phenprocoumaron, warfarin and coumadin) within a time frame of 90 days after discharge from the acute unit or the rehabilitation facility. Additionally documentation for dementia was determined. For this purpose ICD10 codes within the insurance dataset were considered: dementia by Alzheimer’s disease (F00.-, G30.-), vascular dementia (F01.-), secondary dementia (F02.-) and undetermined dementia (F.03).

### Statistical evaluation

Absolute and relative frequencies were calculated based on cross-tables. For testing normal distribution a Kolmogorov-Smirnof Test was applied. Normal distributed variables were tested using a t-test. To compare non-normal distributed data a Mann–Whitney U-test was applied. For comparing frequency data a Fisher’s exact test was used. Factors associated with the outcome in the unadjusted analyses were entered into a logistical analysis for proving parameters in the equation.

## Results

**Figure 1:**
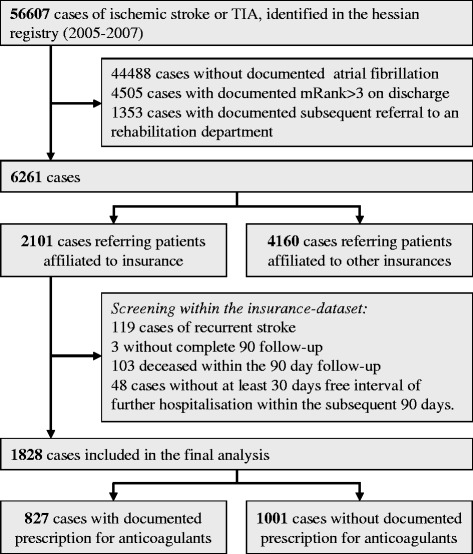
**Patients selection within the stroke registry and after conjunction to the insurance data base.**

Within the GQH dataset 6261 documented cases were identified as stroke patients suffering of atrial fibrillation with a mRS ≤ 3 and no subsequent referral to another department. Out of these 6261 patients, 2101 were identified within the insurance’s dataset. 1828 patients were included in the final analysis as they fulfilled the inclusion criteria (30 days free interval of further hospitalisation and no recurrent stroke with the subsequent 90 days after discharge and complete 90 day follow up). For details see also Figure 1.

**Figure 2:**
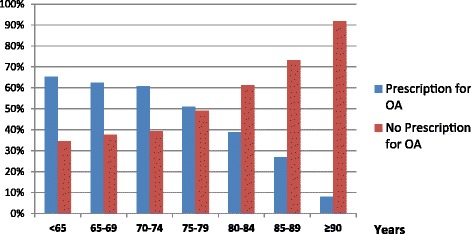
**Distribution of patients (stratified by age) with prescription for OA versus without.**

The mean age of the study population was 77.6 years, 1064 (58.2%) were female. In 639 (35%) patients a transient ischemic attack (TIA) was documented as index stroke event. 1243 (68%) patients were treated in a neurological department and in 205 (11.2%) patients documentation for dementia was evident. In 827 patients (45%) a prescription for OA was identified. With increasing age the proportion of patients treated with OA decreases; while in the age category below 75 years the patients with prescription for OA represented the majority, above this age non-prescription predominated (Figure 2).

Factors such as advanced age, female sex, TIA, worse disability status on discharge, no treatment in a neurological department and documented dementia were associated with no prescription for oral anticoagulants (Table 1). For identifying independent factors, parameters associated in the univariate analysis were entered into a logistical regression analysis (age, sex, disability status as assessed on discharge, treatment in a neurological department and documented dementia). As a cerebrovascular event classified as TIA might directly influence the clinical outcome, which is indicated by the factor mRS, the parameter TIA was not included in the logistical regression analysis. Independently associated with a non-prescription decision for oral anticoagulants in the secondary stroke prevention were: increased age (OR: 0.54, CI: 0.46-0.63; *P* < 0.0001), female sex (OR: 0.77, CI: 0.63-0.94; *P* < 0.011), worse disability status on discharge (OR: 0.88, CI: 0.81-0.96; *P* < 0.006) and documented dementia (OR: 0.54, CI: 0.39-0.73; *P* < 0.001), (Table 2). Treatment in a neurological department was associated with a prescription decision (OR: 1.47, CI: 1.19-1.81; *P* < 0.003), (Table 2).

**Table 1:** **Comparison between patients with prescription for anticoagulants versus without**

	**Total cohort**	**Prescription for OA**	**Non-Prescription for OA**	***P***	OR* (95% CI)
	**(n = 1.828) 100%**	**(n = 827) 45,24%**	**(n = 1.001) 54,76%**		
**Age** (years) median/mean (SD)	77.61 (±8.6)	75.01 (±8.1)	79.75 (±8.5)	< 0.001	
< 65 years	126 (6.9%)	79 (9.6%)	47 (4.7%)	< 0.001	2.14 (1.48-3.11)
65-75 years	537 (29.4%)	323 (39.1%)	214 (21.4%)	< 0.001	2.36 (1.99-2.90)
> 75 years	1165 (63.7%)	425 (51.4%)	740 (73.9%)	< 0.001	0.37 (0.31-0.45)
**Sex**					
Male	764 (41.8%)	398 (48.1%)	366 (36.6%)	< 0.001	ref. category
Female	1064 (58.2%)	429 (51.9%)	635 (63.4%)	< 0.001	0.62 (0.52-0.75)
**Risk factors**					
Hypertension	1444 (79.0%)	660 (79.8%)	784 (78.3%)	0.4	1.09 (0.87-1.37)
Diabetes mellitus	530 (29.0%)	240 (29.0%)	290 (29.0%)	0.9	1.00 (0.82-1.23)
**Diagnosis**					
TIA	639 (35.0%)	260 (31.4%)	379 (37.9%)	< 0.001	0.75 (0.62-0.91)
Previous stroke	433 (23.7%)	179 (21.6%)	254 (25.4%)	0.062	0.81 (0.65-1.01)
**mRS as assessed on discharge**					
0	557 (30.5%)	282 (34.1%)	275 (27.5%)	0.002	1.37 (1.19-1.67)
1	478 (26.1%)	226 (27.3%)	252 (25.2%)	0.3	1.18 (0.91-1.38)
2	403 (22.0%)	174 (21.0%)	229 (22.9%)	0.3	0.90 (0.72-1.12)
3	390 (21.3%)	145 (17.5%)	245 (24.5%)	< 0.001	0.66 (0.52-0.82)
Median, range	1 (0–3)	1 (0–3)	1 (0–3)		
**Treatment in a neurological department**	1243 (68.0%)	611 (73.9%)	632 (63.1%)	< 0.001	1.65 (1.35-2.02)
**Comorbidities occurring while hospitalisation**					
Intracranial bleeding	8 (0.4%)	5 (0.6%)	3 (0.3%)	0.3	2.02 (0.48-8.49)
Extracranial bleeding	7 (0.4%)	4 (0.5%)	3 (0.3%)	0.5	1.61 (0.36-7.24)
Recurrent stroke/TIA	17 (0.9%)	12 (1.5%)	5 (0.5%)	0.04	2.93 (1.03-8.36)
Seizure	6 (0.3%)	0 (0%)	6 (0.6%)	0.03	not applicable
Pneumonia	42 (2.3%)	18 (2.2%)	24 (2.4%)	0.8	0.91 (0.49-1.69)
**Documented dementia within the insurance database** ^**#**^	241 (13.2%)	67 (7.2%)	174 (14.2%)	< 0.001	0.42 (0.31-0.56)

*Value calculated in a **Chi** Square Test of Independence.

^#^dementia documented within the insurance database on discharge or within 90 days after discharge; considered were the following ICD10 codes: dementia by Alzheimer’s disease (F00.-), vascular dementia (F01.-, G30), secondary dementia (F02.-) and undetermined dementia (F.03).

AK refers to anticoagulants.

TIA refers to transient ischaemic attack.

mRS refers to modified Rankin scale.

**Table 2:** **Logistical regression analysis (parameters associated with prescription/non-prescription in the univariate analysis (p < 0.05) were considered)**

	**OR (95% CI)**	***P***
Age (in categories)		< 0.001
Higher age	0.54 (0.46-0.63)	
Younger age	1.86 (1.58-2.19)	
Sex		0.011
Female	0.77 (0.63-0.94)	
Male	1.29 (1.06-1.58)	
mRS as assesses on Discharge (in categories)		0.006
Higher disability	0.88 (0.81-0.96)	
Lower disability	1.13 (1.04-1.38)	
Treatment in a neurological department		0.003
Yes	1.47 (1.19-1.81)	
No	0.68 (0.55-0.84)	
Documented dementia within the insurance database^#^		< 0.001
Yes	0.54 (0.39-0.73)	
No	1.86 (1.37-2.53)	

^#^dementia documented within the insurance database on discharge or within 90 days after discharge; considered were the following ICD10 codes: dementia by Alzheimer’s disease (F00.-), vascular dementia (F01.-, G30), secondary dementia (F02.-) and undetermined dementia (F.03).

## Discussion

In studying a group of stroke patients with AF with a best case scenario, we detected a low rate (45%) of prescriptions for OA. This is a surprising result, considering the selected patients were most suitable for OA therapy; after the acute stroke they all had a favorable disability status (mRS ≤ 3 as assessed on discharge). With increasing age the proportion of patients treated with OA decreased (Figure 2). While advanced age, female sex, worse disability status and dementia were associated with a non-prescription, treatment in a specialized neurological department facilitated the therapeutic decision for OA in the secondary prevention.

Considering the low rate of prescriptions for OA in our study and the bulk of evidence, indicating the necessity for this therapy, the situation in the broad care delivery seems to follow particular rules [2,4,5,14,15]. Even though the evidence provided in the Birmingham Atrial Fibrillation Treatment of the Aged (BAFTA) indicates superiority of OA against aspirin in elderly patients, increased age was associated with non-prescription for OA [8]. However, in comparison to recent trials, which investigated the efficacy of OA in AF, patients selected for OA in our study were older (median 76 versus 70–71 years) [2,4,14]. Nevertheless, 55% of the patients seen were not prescribed OA which might be explained by the generally advanced age range (median 79 years) in patients encountered in daily practice.

A further aspect which needs to be taken into consideration is the risk for bleeding. This risk increases with increasing age [16,17]. Particularly in the case of intracranial hemorrhages, the risk increases substantially for patients over 75 years in age [15,18]. This clearly influences the practitioner’s decision-making when selecting treatment options. Whereas, in the age category from 75 to 79 years half of the patients are treated by OA, in individuals ≥80 years the decision for an OA is considerably less frequent (Figure 2). Weighing all the arguments for OA for secondary stroke prevention in relation to the safety concerns, the reluctance to prescribe OA for elderly patients is understandable. A clinician’s treatment decisions are guided by balancing effectiveness against risk to harm. As indicated by our results, safety concerns prevail. In order to minimize difficulties in the adequate selection of patients for OA, clear evidence on efficacy and safety among patients occurring in the real world are required. Future studies should address treatment decisions for elderly patient care. Furthermore, studies proving treatment effects in the secondary stroke prevention are necessary.

However, age was not the only determinant which contributed to the low usage rate of OA among stroke patients with AF in the clinical practice. An additional independent factor in facilitating the prescription for OA therapy was the treatment of the acute event in a specialized neurological department. Therapeutic recommendations from specialized departments seem to be of considerable relevance for the subsequent secondary stroke prevention emphasizing the need to establish such units. Furthermore, specific educational programs for general practitioners and specialists who are involved in the treatment of stroke patients with AF, appears necessary.

Concerning the disability status, there is no conclusive reason for not prescribing OA in our study. All selected patients were not severely disabled (mRS ≤ 3 as assessed on discharge), which implies the patient’s ability to walk independently as well as minor assistance needed in coping with daily tasks. However, even in this range a slight deterioration results in the decision for an alternative therapy to OA.

The low rate of OA prescriptions in patients with documented dementia is not surprising, given to the need for monitoring this treatment precisely. However, there is no credible argument to withhold an effective therapy to patients with cognitive decline. Given the availability of newer anticoagulants with slightly less treatment monitoring requirements, these patients can benefit from OA usage in the future.

The identified factors might not be sufficient to entirely explain the low rate of patients selected for OA. The influence of further factors including cerebral imaging findings, the individual preference or acquisition of medication other than by prescription was not captured in our study. Thus, their relevance on the decision for or against OA remains unclear.

## Conclusion

In the delivery of patient care, more than half of the patients with AF after stroke did not receive OA medication; 50% of these patients were above 81 years of age. In balancing effectiveness versus risk to harm, safety concerns prevail regarding therapeutic decisions. This is predominantly due to the high proportion of elderly patients receiving care. The factor of advanced age is correlated with a decision against OA. Patients with dementia were also less likely to receive OA. Even though all our subjects were only slightly affected by stroke (mRS ≤ 3), we noticed a decreased use of OA as there was an increased worsening of the disability. In contrast, patients treated in specialized neurological departments were more likely to receive OA as a treatment option. As a result, it would be beneficial providing additional education programs to non-specialists treating stroke patients make them more aware of its clinical benefits over other treatment options.

## Abbreviations

OA: Oral anticoagulation
BAFTA: Birmingham atrial fibrillation treatment of the aged
TIA: Transient ischemic attack
GQH: Register of the institute of quality assurance hesse (Geschäftsstelle für Qualitätssicherung)
AF: Atrial fibrillation
mRS: Modified rankin scale

## References

[1] **Secondary prevention in non-rheumatic atrial fibrillation after transient ischaemic attack or minor stroke. EAFT (European Atrial Fibrillation Trial) Study Group.** *Lancet* 1993, **342:**1255–1262. 7901582

[2] Connolly SJ, Ezekowitz MD, Yusuf S, Eikelboom J, Oldgren J, Parekh A, Pogue J, Reilly PA, Themeles E, Varrone J, Wang S, Alings M, Xavier D, Zhu J, Diaz R, Lewis BS, Darius H, Diener HC, Joyner CD, Wallentin L (2009). Dabigatran versus warfarin in patients with atrial fibrillation. N Engl J Med.

[3] Granger CB, Alexander JH, McMurray JJ, Lopes RD, Hylek EM, Hanna M, Al-Khalidi HR, Ansell J, Atar D, Avezum A, Bahit MC, Diaz R, Easton JD, Ezekowitz JA, Flaker G, Garcia D, Geraldes M, Gersh BJ, Golitsyn S, Goto S, Hermosillo AG, Hohnloser SH, Horowitz J, Mohan P, Jansky P, Lewis BS, Lopez-Sendon JL, Pais P, Parkhomenko A, Verheugt FW (2011). Apixaban versus warfarin in patients with atrial fibrillation. N Engl J Med.

[4] Hankey GJ, Patel MR, Stevens SR, Becker RC, Breithardt G, Carolei A, Diener HC, Donnan GA, Halperin JL, Mahaffey KW, Mas JL, Massaro A, Norrving B, Nessel CC, Paolini JF, Roine RO, Singer DE, Wong L, Califf RM, Fox KA, Hacke W (2012). Rivaroxaban compared with warfarin in patients with atrial fibrillation and previous stroke or transient ischaemic attack: a subgroup analysis of ROCKET AF. Lancet Neurol.

[5] van Walraven C, Hart RG, Singer DE, Laupacis A, Connolly S, Petersen P, Koudstaal PJ, Chang Y, Hellemons B (2002). Oral anticoagulants vs aspirin in nonvalvular atrial fibrillation: an individual patient meta-analysis. JAMA.

[6] Olesen JB, Lip GY, Hansen ML, Hansen PR, Tolstrup JS, Lindhardsen J, Selmer C, Ahlehoff O, Olsen AM, Gislason GH, Torp-Pedersen C (2011). Validation of risk stratification schemes for predicting stroke and thromboembolism in patients with atrial fibrillation: nationwide cohort study. BMJ.

[7] Lip G, Nieuwlaat R, Pisters R, Lane D, Crijns H (2009). Refining clinical risk stratification for predicting stroke and thromboembolism in atrial fibrillation using a novel risk factor based approach: The Euro Heart Survey on Atrial Fibrillation. Chest.

[8] Mant J, Hobbs FD, Fletcher K, Roalfe A, Fitzmaurice D, Lip GY, Murray E (2007). Warfarin versus aspirin for stroke prevention in an elderly community population with atrial fibrillation (the Birmingham Atrial Fibrillation Treatment of the Aged Study, BAFTA): a randomised controlled trial. Lancet.

[9] Russolillo A, Di Minno MN, Tufano A, Prisco D, Di MG (2012). Filling the gap between science & clinical practice: prevention of stroke recurrence. Thromb Res.

[10] Dinh T, Nieuwlaat R, Tieleman RG, Buller HR, van Charante NA, Prins MH, Crijns HJ (2007). Antithrombotic drug prescription in atrial fibrillation and its rationale among general practitioners, internists and cardiologists in The Netherlands–The EXAMINE-AF study. A questionnaire survey. Int J Clin Pract.

[11] Nieuwlaat R, Capucci A, Lip GY, Olsson SB, Prins MH, Nieman FH, Lopez-Sendon J, Vardas PE, Aliot E, Santini M, Crijns HJ (2006). Antithrombotic treatment in real-life atrial fibrillation patients: a report from the Euro Heart Survey on Atrial Fibrillation. Eur Heart J.

[12] Foerch C, Misselwitz B, Sitzer M, Berger K, Steinmetz H, Neumann-Haefelin T (2005). Difference in recognition of right and left hemispheric stroke. Lancet.

[13] Stolz E, Hamann GF, Kaps M, Misselwitz B (2011). Regional differences in acute stroke admission and thrombolysis rates in the German federal state of Hesse. Dtsch Arztebl Int.

[14] Diener HC, Connolly SJ, Ezekowitz MD, Wallentin L, Reilly PA, Yang S, Xavier D, Di PG, Yusuf S (2010). Dabigatran compared with warfarin in patients with atrial fibrillation and previous transient ischaemic attack or stroke: a subgroup analysis of the RE-LY trial. Lancet Neurol.

[15] Fang MC, Chang Y, Hylek EM, Rosand J, Greenberg SM, Go AS, Singer DE (2004). Advanced age, anticoagulation intensity, and risk for intracranial hemorrhage among patients taking warfarin for atrial fibrillation. Ann Intern Med.

[16] Hylek EM, Evans-Molina C, Shea C, Henault LE, Regan S (2007). Major hemorrhage and tolerability of warfarin in the first year of therapy among elderly patients with atrial fibrillation. Circulation.

[17] Pisters R, Nieuwlaat R, de Vos CB, Crijns HJ (2009). Comprehensive upstream treatment for atrial fibrillation, when and how?. Europace.

[18] Eikelboom JW, Wallentin L, Connolly SJ, Ezekowitz M, Healey JS, Oldgren J, Yang S, Alings M, Kaatz S, Hohnloser SH, Diener HC, Franzosi MG, Huber K, Reilly P, Varrone J, Yusuf S (2011). Risk of bleeding with 2 doses of dabigatran compared with warfarin in older and younger patients with atrial fibrillation: an analysis of the randomized evaluation of long-term anticoagulant therapy (RE-LY) trial. Circulation.

